# Nutritional Advantages of Walnut (*Juglans regia* L.) for Cardiovascular Diseases: A Comprehensive Review

**DOI:** 10.1002/fsn3.4526

**Published:** 2024-12-30

**Authors:** Mostafa Rashki, Mahboobeh Ghasemzadeh Rahbardar, Mohammad Hossein Boskabady

**Affiliations:** ^1^ Applied Biomedical Research Center Mashhad University of Medical Sciences Mashhad Iran; ^2^ Department of Physiology, Faculty of Medicine Mashhad University of Medical Sciences Mashhad Iran

**Keywords:** blood pressure, inflammation, *Juglans regia*, oxidative stress, stroke, thrombosis, walnut

## Abstract

Cardiovascular diseases (CVDs) remain one of the leading causes of morbidity and mortality worldwide. In recent years, the potential role of dietary interventions in preventing and managing CVDs has gained significant attention. Among these dietary components, walnuts (*Juglans regia* L.) have emerged as a promising candidate due to their unique nutrient profile and potential cardiovascular benefits. This review aims to provide a comprehensive analysis of the existing literature on the role of walnuts in cardiovascular health. Using databases from Scopus, Google Scholar, and PubMed, the most relevant in vitro, in vivo, and clinical trial research has been collected from the time of inception until 2024. Several studies have shown that walnut consumption has a positive effect on a variety of cardiovascular risk factors. Walnut bioactive ingredients, including omega‐3 fatty acids, antioxidants, fiber, and polyphenols, have been demonstrated to improve lipid profiles, blood pressure, endothelial function, inflammation, oxidative stress, and thrombosis. These processes all contribute to the possible cardioprotective properties of walnuts. Epidemiological and clinical research indicates that daily walnut consumption can reduce the risk of CVDs like coronary heart disease and stroke. Walnuts may aid in managing CVDs through mechanisms such as enhancing lipid profiles, reducing inflammation, and improving overall cardiovascular function. This review highlights the potential role of walnuts as a dietary strategy for the prevention and management of CVDs. Further understanding of the mechanisms and long‐term effects of walnut consumption is crucial for optimizing their therapeutic potential and integrating them into clinical practice. Future research should focus on elucidating specific dose–response relationships and exploring the synergistic effects of walnuts in combination with other dietary and lifestyle interventions.

## Graphical abstract

The study discusses the unique nutrient profile of walnuts and their potential cardiovascular benefits. We have carefully reviewed and summarized the available literature on the effects of walnut consumption on various cardiovascular risk factors such as lipid profiles, blood pressure, endothelial function, inflammation, oxidative stress, and thrombosis. Additionally, we have examined the epidemiological and clinical evidence supporting the association between walnut consumption and a reduced risk of developing CVDs, including coronary heart disease and stroke.

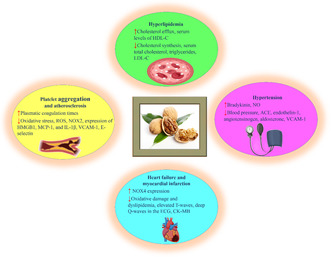

**Abbreviations**
ABCG1: ATP‐binding cassette subfamily G member 1
ACC: Acetyl‐CoA carboxylase
ACE: Angiotensin‐converting enzyme
ACOX1: Acyl‐CoA oxidase 1
ALT: alanine aminotransferase
Apo: Apolipoprotein
AST: aspartate aminotransferase
BP: blood pressure
CAT: Catalase
ChREBP: carbohydrate response element‐binding protein
CK‐MB: creatine kinase‐MB
CPK: creatine phosphokinase
CPT1A: Carnitine palmitoyltransferase 1A
cTnI: cardiac troponin I
CYP51: Cytochrome P450 51
CYP7A1: Cytochrome P450 7A1
DBP: diastolic blood pressure
ECG: electrocardiogram
FAS: Fatty acid synthase
GPx: glutathione peroxidase
GSH: glutathione
HDL‐C: High‐density lipoprotein cholesterol
HMGB1: high mobility group box 1
HMGCR: 3‐hydroxy‐3‐methylglutaryl‐coenzyme A reductase
HMGR: Hydroxymethylglutaryl‐coenzyme A reductase
IL‐1β: interleukin‐1β
LCAT: Lecithin‐cholesterol acyltransferase
LDH: lactate dehydrogenase
LDL‐C: Low‐density lipoprotein cholesterol
MAP: mean arterial pressure
MCP‐1: monocyte chemoattractant protein‐1
MDA: Malondialdehyde
mRNA: Messenger RNA
NO: nitric oxide
NOX2: NADPH oxidase 2
PAI‐1: Plasminogen activator inhibitor‐1
PPARα: Peroxisome proliferator‐activated receptor alpha
ROS: reactive oxygen species
SBP: systolic blood pressure
SCD‐1: Stearoyl‐CoA desaturase‐1
SOD: Superoxide dismutase
SREBP: Sterol regulatory element‐binding protein
TC: total cholesterol
TG: triglycerides
TNF‐α: tumor necrosis factor‐alpha
tPA: Tissue plasminogen activator
VCAM‐1: vascular cell adhesion molecule 1
VLDL‐C: Very low‐density lipoprotein cholesterol

## 1 Introduction

Cardiovascular diseases (CVDs) are a major global health challenge, accounting for a significant percentage of deaths and disabilities globally (Mensah, Roth, and Fuster 2019). These disorders, which include coronary heart disease, stroke, and hypertension (Gupta et al. 2008), have complicated underlying processes that include inflammation, oxidative stress, endothelial dysfunction, and dyslipidemia (Medina‐Leyte et al. 2021). Current CVD treatment options often involve pharmaceutical products that target these pathways, such as lipid‐lowering statins, antihypertensive medicines, and antiplatelet medications (Hua et al. 2020; Coca et al. 2020). However, these treatments can have adverse effects and limits, emphasizing the need for alternate methods and the development of novel therapeutic agents (Shaito et al. 2020).

Cardiovascular diseases present a significant concern, as evidenced by epidemiological statistics. According to the World Health Organization (WHO), CVDs caused about 17.9 million deaths worldwide in 2019, accounting for 32% of all fatalities. CVDs impact people from every socioeconomic level, including low‐ and middle‐income nations, which account for over 75% of CVD‐related fatalities (World Health Organization 2021). These findings highlight the critical need to investigate novel techniques for prevention, treatment, and controlling CVDs.

In this context, exploring the potential of natural therapeutic interventions is crucial. Traditional medicine has long been recognized for its ability to manage and treat a variety of illnesses including, cardiovascular disease. Many herbs or their active components have been illustrated to have cardioprotective effects including, *Rosa damascena* (Boskabady et al. 2011), *Nigella sativa* (Fadishei et al. 2021), *Solanum melongena* (Yarmohammadi, Ghasemzadeh Rahbardar, and Hosseinzadeh 2021), alpha‐mangostin (Eisvand et al. 2022; Ardakanian et al. 2022), rosmarinic acid (Rahbardar et al. 2022), carnosic acid (Ghasemzadeh Rahbardar et al. 2024), and alpha‐lipoic acid (Najafi et al. 2022).

*Juglans regia* L. (*J. regia*), or walnut, is a tree nut native to southeastern Europe, eastern Asia, and northern America (Aradhya et al. 2017; Pollegioni et al. 2017). Walnuts have been an important element of the human diet throughout history. The kernel of the walnut fruit has been cherished for its rich flavor and nutritional advantages, whether consumed fresh, roasted, or added to various confectionaries. Walnuts are frequently coupled with almonds, dates, and raisins in traditional Middle Eastern desserts (Taha and Al‐wadaan 2011; Ghasemzadeh Rahbardar and Hosseinzadeh 2024). Because of its many uses in traditional medicine worldwide, *J. regia* leaves are used for a variety of ailments. These include antibacterial, antihelmintic, astringent, keratolytic, antidiarrheal, hypoglycemic, depurative, tonic, and carminative effects. They have also been used to treat stomachaches, colds, and sinusitis (Taha and Al‐wadaan 2011; Mouhajir et al. 2001; Jaiswal and Tailang 2017). Fresh leaves are applied topically to the body or forehead in Turkish folk medicine to lower fever or to swollen joints to relieve rheumatic pain (Yeşilada 2002). In addition, Iranian medicine has long used the *J. regia* kernel to treat inflammatory bowel disease (Rahimi, Shams‐Ardekani, and Abdollahi 2010). Avicenna mentioned that different parts of the *J. regia* tree, such as the leaves, bark, and fruit, are beneficial for treating ailments like diarrhea, skin conditions, and respiratory issues (Avicenna 2019). Moreover, in Makhzan al‐Adwiya, Mohammad Hossein Aghili Khorasani emphasized the advantageous effects of *J. regia* on the digestive system, as well as its potential benefits for skin conditions, diabetes, and wound healing (Khorasani n.d.). In another ancient book named “Tohfat al‐Momenin” the therapeutic uses of walnut for conditions such as constipation, diarrhea, and stomach disorders were declared. It also mentions the application of walnut oil for skin‐related issues (Momen Tonekaboni n.d.). The leaves are used in Palestine to treat diabetes and asthma (Kaileh et al. 2007). The mentioned medicinal plant is used topically to treat a variety of ailments, including scrofula, persistent eczema, excessive hand and foot sweating, and skin irritation. Its leaves are applied topically to relieve sunburn, dandruff, itchy scalp, and minor burns. Also, they act as an adjunctive emollient in the treatment of various skin disorders. This plant fruit is noteworthy for its strong osteoblastic activity and notable anti‐atherogenic potential, which add to the benefits of a walnut‐rich diet in terms of heart protection and preventing bone loss. This medicinal plant's exocarp from immature green fruit, branches, and bark has long been utilized in Chinese medicine to cure lung, liver, and stomach cancers. It is used by traditional healers in northern Mexico to protect the liver. To clean teeth, the bark is used to make miswaks. Bark paste is used in Nepal to treat skin conditions such as toothaches, arthritis, and the growth of hair. The seed coat also aids in the healing of wounds. Lastly, the *J. regia* shell is used in Calabrian traditional medicine to cure malaria (Taha and Al‐wadaan 2011; Jaiswal and Tailang 2017).

The kernels of walnut are a nutrient‐dense food because of their high levels of proteins (Özcan 2009), unsaturated fatty acids (Liu et al. 2019), minerals (Cosmulescu et al. 2009), and polyphenols (Sánchez‐González et al. 2017). Sterols, flavonoids, phenolic acids, pectic compounds, and other polyphenols are abundant in these nuts, contributing to their overall nutritional profile (Taha and Al‐wadaan 2011). Unsaturated fatty acids in walnut have been shown to provide a variety of health advantages, such as cardioprotective effects including lowering blood cholesterol levels and providing antioxidant protection (Richard et al. 2008). Furthermore, investigations have demonstrated that walnut protein hydrolysates can scavenge free radicals and prevent lipid peroxidation, emphasizing their potential antioxidant properties (Jahanbani et al. 2016). In addition, walnut proteins have been revealed to be a useful source of bioactive peptides with hypocholesterolemic and angiotensin‐converting enzyme (ACE) inhibitory effects (Liu et al. 2019, 2013). Besides, omega‐3 fatty acids present in walnuts have been demonstrated to lower triglyceride levels (Ashraf et al. 2020). Table 1 presents phytochemicals from other parts of the walnut tree, such as the internal septum, husk, and walnut leaf, some of which have shown promising cardioprotective effects, including catechins (Ferenczyová et al. 2021), proanthocyanidins (Wang et al. 2020), phenolic compounds (Toma et al. 2020), gallic acid (Akbari 2020), quercitrin (Liu et al. 2024), protocatechuic acid (Li et al. 2020), vanillic acid (Lashgari et al. 2023), ethyl gallate (Liu, Liu, et al. 2021), and dihydroquercetin (Usmanov et al. 2021).

**TABLE 1:** Chemical composition of different parts of the *J. regia* tree.

Part of *J. regia* tree	Compounds	References
Septum	Lipids Low‐molecular‐mass phenolic carboxylic acids Phenolic aldehydes Catechins Proanthocyanidins Oligomeric and polymeric phenolic substances Gallic acid Dihydrophaseic acid Blumenol B Quercitrin Protocatechuic acid Taxifolin‐3‐O‐α‐L‐arabinofuranoside ρ‐Hydroxybenzoic acid Vanillic acid Ethyl gallate Dihydroquercetin (4s)‐4‐hydroxy‐1‐tetralone (+)‐dehydrovomifoliol (6R,9S)‐9‐hydroxymegastigman‐4‐en‐3‐one	Ghiravani et al. (2020), Bezhuashivili and Kurashvili (1998), Wang et al. (2017)
Green husk	Ethylene oxide 2‐Formylhistamine Cyclohexane Acetanone Cyclotrisiloxane, hexamethyl N‐ethyl‐1,3‐dithiosoindoline Cyclotetrasixane,octamethyl N‐methyl‐propylamine Cyclopentaasiloxane,decamethyl 2,4‐di(trimethylsiloxy)‐6,7‐ (methyl lenedioxy) 11H‐Dibenzo p‐Meth‐1‐en‐3,1,semicarbazone Pentadecone 6‐Aza‐5,7,12,14‐tetrathiapentacene	Keskin, Ceyhan, and Ugur (2012)
Leaves	Ethylene oxide Cyclohexane Carbonic acid Cyclotrisiloxane, hexamethyl Acetic acid, ethyl ester Ethanol Cyclotetrasixane,octamethyl 2‐Pentanone Methane Spiro(cyclopenta) Cyclopentasiloxane,decamethyl 6‐Aza‐5,7,12,14‐Tetrathiapentacene 2,4‐di(trimethylsiloxy) Acetic acid 1‐Pentene,1,3diphenyl‐1 Methoxycarbonyl Benzaldehyde,2,4‐bis Dimethylamine Benzoic acid	Keskin, Ceyhan, and Ugur (2012)

The aim of this review is to provide a comprehensive analysis of the current literature on the role of walnuts in cardiovascular health. By gathering the available evidence, this review seeks to bridge the gap between existing knowledge and clinical approaches, highlighting the potential of walnuts as a natural therapeutic intervention for CVDs. The significance of this review lies in its potential to contribute to the development of novel treatment strategies, offering a complementary approach to conventional pharmacological interventions. By exploring the pharmacological effects of walnuts and their potential mechanisms of action, this review aims to provide valuable insights for researchers, healthcare professionals, and individuals seeking to optimize their cardiovascular health. Moreover, by emphasizing the potential of walnuts as a complementary approach to conventional pharmacological interventions, this review may contribute to the development of novel treatment strategies.

## 2 Methods

To conduct this research, several databases, including Scopus, Google Scholar, and PubMed, were utilized. The search encompassed publications from in vitro studies, in vivo studies, and clinical trials, with data collection up until the end of December 2023. The search terms used included “walnut”, “*Juglans regia* L.”, “hypertension”, “anti‐hypertensive”, “hyperlipidemia”, “atherosclerosis”, “coronary artery disease”, “myocardial infarction”, “heart attack”, “stroke”, “heart failure”, “arrhythmias”, “peripheral artery disease”, “valvular heart disease”, “cardiovascular diseases”, “blood pressure”, “thrombosis”, “endothelial function”, “coronary heart disease”, “cerebrovascular disease”, “peripheral arterial disease”, “rheumatic heart disease”, “congenital heart disease”, “deep vein thrombosis and pulmonary embolism”, “heart attacks”, and “strokes”.

For searching published paper, the inclusion were

- Articles investigating the effects of walnut on cardiovascular diseases.
- Studies conducted on human subjects, animal models, or in vitro models.
- Articles written in English.
- Articles published in peer‐reviewed journals.

Also, the following types of articles were excluded in this review:

- Non‐English articles.
- Studies that did not focus on cardiovascular diseases or the effects of walnut.
- Non‐peer‐reviewed articles, such as conference abstracts, letters, and editorials.

**FIGURE 1:**
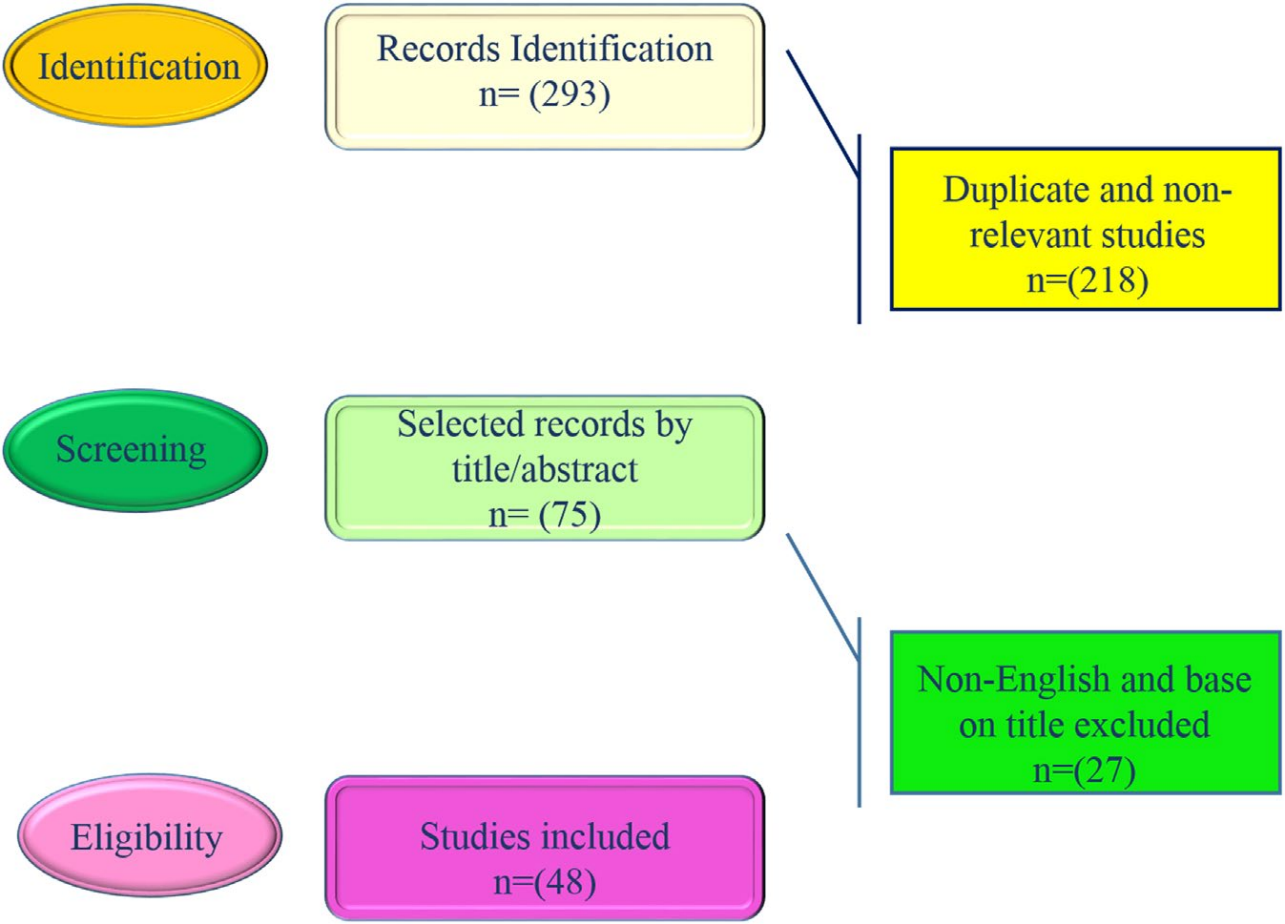
The chart of search strategy.

Totally 293 articles were identified which 218 were non‐relevant or duplicated and 27 were published in non‐English language, thus 48 papers were included in the review process (Figure 1).

## 3 The *Juglans regia* L. Effects on CVDs

### 3.1 Hyperlipidemia

Hyperlipidemia is a medical disorder characterized by elevated levels of lipids in the circulation (Jalali and Ghasemzadeh 2022). It plays a part in the development and progression of CVDs. The relationship between hyperlipidemia and CVDs is well‐established, with several studies determining its role in the pathogenesis of atherosclerosis and its related complications (Oskouei, Ghasemzadeh Rahbardar, and Hosseinzadeh 2023; Alloubani, Nimer, and Samara 2021). Low‐density lipoprotein cholesterol (LDL‐C), known as “bad” cholesterol, promotes the development of atherosclerotic plaques within artery walls (Linton et al. 2019). In addition, hyperlipidemia is related to lower levels of high‐density lipoprotein cholesterol (HDL‐C), also known as “good” cholesterol, which impairs the reverse cholesterol transport mechanism and accelerates atherosclerosis (Ouimet, Barrett, and Fisher 2019). The accumulation of lipid‐rich plaques in the arterial walls can ultimately result in life‐threatening conditions such as myocardial infarction, stroke, and peripheral artery disease (Attar et al. 2019).

Several factors contribute to the underlying mechanisms of hyperlipidemia and the induction of CVDs. Genes encoding 3‐hydroxy‐3‐methylglutaryl‐CoA reductase (HMGCR), sterol regulatory element‐binding protein (SREBP)‐2, and sterol 14α‐demethylase cytochrome P450 (CYP51) are involved in cholesterol synthesis. As a major regulatory enzyme controlling endogenous cholesterol synthesis, HMGCR is the rate‐limiting enzyme in the de novo synthesis of cholesterol (Ma et al. 2019). SREBP‐2 is a transcription factor that regulates cholesterol biosynthesis (Tao et al. 2013). CYP51 is an enzyme involved in the synthesis of cholesterol precursors (Lorbek, Lewinska, and Rozman 2012). Dysregulation of these genes can result in increased cholesterol levels, contributing to hyperlipidemia and atherosclerosis (Chmielewski et al. 2007; Gao et al. 2022a).

In addition, genes related to fatty acid oxidation and lipid metabolism, such as peroxisome proliferator‐activated receptor α (PPARα), acyl coenzyme A oxidase (ACOX)1, and carnitine palmitoyltransferase (CPT)1A, play significant roles in the development of hyperlipidemia and CVDs (Shimoda et al. 2009; Takei et al. 2017). PPARα is a transcription factor that regulates the expression of genes involved in fatty acid oxidation (van Raalte et al. 2004). ACOX1 and CPT1A are enzymes responsible for the breakdown of fatty acids for energy production (Li et al. 2021). Dysregulation of these genes can disrupt lipid metabolism, contributing to the development of hyperlipidemia and CVDs (Shimoda et al. 2009; Li et al. 2015; Yuan et al. 2020). Proteins involved in the regulation of blood clotting and fibrinolysis, such as plasminogen activator inhibitor‐1 (PAI‐1) and tissue plasminogen activator (tPA), also play a role in hyperlipidemia and the progression of CVDs. Elevated levels of PAI‐1 can impair fibrinolysis, leading to increased thrombotic events and the progression of atherosclerosis (Sillen and Declerck 2020). Conversely, tPA promotes the breakdown of blood clots (El‐Sherbiny, Elkholi, and Yacoub 2014). Imbalances in the regulation of these proteins can contribute to the development of CVDs (Morrow, Whyte, and Mutch 2021).

Furthermore, oxidative stress, characterized by an imbalance between reactive oxygen species (ROS) production and antioxidant defenses, plays a significant role in the development of hyperlipidemia and CVDs (Guo et al. 2020). Oxidative stress promotes lipid peroxidation, inflammation, endothelial dysfunction, and the formation of atherosclerotic plaques (Marchio et al. 2019).

The subsequent part will include investigations on the effects of *J. regia* on hyperlipidemia.

#### 3.1.1 In Vitro

Treating HepG2 cells with *J. regia* oil resulted in a notable reduction in cholesterol synthesis, which was achieved by decreasing the expression of specific genes involved in cholesterol production, namely HMGCR, SREBP‐2, and CYP51. Additionally, *J. regia* oil increased cholesterol efflux by upregulating the expression of ATP‐binding cassette sub‐family G member 1 (ABCG1), leading to a significant decrease in total cholesterol and triacylglycerol levels. These beneficial effects were attributed to the presence of phytosterols and total phenolic compounds in *J. regia* oil, which played a role in lowering cholesterol (Gao et al. 2022a).

#### 3.1.2 Studies Including Both In Vitro and In Vivo Sections

Exposing HepG2 cells to a dried kernel pellicles of *J. regia* polyphenol‐rich extract (50% ethanol) increased messenger ribonucleic acid (mRNA) expressions of PPARα, ACOX1, and CPT1A. Besides, treating these cells with Tellimagrandin, a polyphenolic constituent of polyphenol‐rich extract from *J. regia*, boosted the expression of ACOX1. The findings of the in vivo part of the study illustrated that the administration of a polyphenol‐rich extract from *J. regia* to mice with hypertriglyceridemia increased the mRNA expressions of PPARα and ACOX1, and reduced the levels of triglycerides (Shimoda et al. 2009).

#### 3.1.3 In Vivo

A study in 1986 found that both the *J. regia* kernel and its oil, whether unrefined or refined, were effective in reducing serum cholesterol, triglycerides, phospholipids, and total lipids in hypercholesterolemic rats. The efficacy of the *J. regia* kernel was attributed to its oil content, which contains approximately 70% polyunsaturated fatty acids. The study also showed that the unsaponifiable matter and sterol contents of the oil were not responsible for its effectiveness. Additionally, the hypolipidemic effect observed with *J. regia* kernel or its oil was not due to differences in fecal cholesterol. Furthermore, a blend of vanaspati (partially hydrogenated vegetable oil) and *J. regia* oil in equal proportions was as effective as *J. regia* kernel and its oil, highlighting the potential value of *J. regia* kernel as a food material in reducing serum lipid constituents (George et al. 1986). The supplementation of an alcoholic extract of leaves of *J. regia* to diabetic rats attenuated the plasma levels of LDL, total cholesterol, and triglycerides, but increased HDL‐C (Mohammadi et al. 2011). It has been indicated that treating diabetic rats with whole *J. regia* reduced atherosclerosis risk factors by augmenting the amounts of HDL‐C and PPARα but decreasing the levels of cholesterol, LDL‐C, VLDL‐C, triglycerides, and SREBP‐1c protein levels (Ebrahim Abbasi, Arash Noori, and Ali 2012). The oral administration of an ethanolic extract of the internal septum of *J. regia* fruit to diabetic rats caused a significant decrease in triglycerides, total cholesterol, and LDL (Ghiravani et al. 2016). Adding *J. regia* powder to the food of rats with a high‐fat diet and hyperlipidemia increased blood anti‐oxidant capacity, HDL‐C, and lessened levels of LDL‐C (Abrar et al. 2019). It has been reported that treating pregnant diabetic rats with *J. regia* nut oil‐derived polyunsaturated fatty acid resulted in enhanced HDL‐C amounts in plasma, beside attenuated levels of total cholesterol and triglycerides, as well as decreased mRNA expression of LDL‐C, acetyl‐coenzyme A carboxylase (ACC), fatty acid synthase (FAS), stearoyl‐CoA desaturase‐1 (SCD‐1), and SREBP‐1 (Sun et al. 2020). Similarly, it has been demonstrated that the supplementation of an ethanolic extract of *J. regia* leaves to hypercholesterolemic rats increased HDL‐C and attenuated the levels of triglycerides, LDL, and the atherogenic index measured by serum total cholesterol/HDL and LDL/HDL ratios (Azhar et al. 2021). Supplementing *J. regia* kernel meal peptides to hyperlipidemic rats with a high‐fat diet reduced the atherosclerosis index through enhancing HDL‐C, lecithin cholesterol acyl transferase (LCAT), cholesterol 7 alpha‐hydroxylase (CYP7A1), and lowering total cholesterol, triglycerides, and LDL‐C, Apolipoprotein (Apo)‐B, Apo‐A1, FAS, and 3‐hydroxy‐3‐methylglutaryl‐coenzyme A (HMGR) (Yang et al. 2021). The administration of a methanolic extract of *J. regia* husk to hypercholesterolemic rats with a high‐fat diet increased the levels of HDL but reduced the amounts of cholesterol, LDL, and triglycerides (Rozha et al. 2021). Moreover, *J. regia* (whole fruit without shells) powder decreased VLDL in rats with a high‐fat diet (Sangi, Shamim, and Khan 2022). The administration of defatted *J. regia* kernel powder extract to hyperlipidemic rats reduced plasma total cholesterol, triglyceride, and LDL‐C (Xu et al. 2022). The administration of an ethanolic extract of *J. regia* fruit diaphragm to hyperlipidemic rats increased HDL‐C, catalase (CAT), and superoxide dismutase (SOD) amounts in the blood. It also decreased the blood amounts of total cholesterol, triglyceride, and malondialdehyde (MDA) (Palabiyik et al. 2023).

#### 3.1.4 Clinical Trial

It has been shown that substituting *J. regia* (raw, shelled) for monounsaturated fat (*J. regia* replaced about 35% of the energy achieved from monounsaturated fat) lowered total cholesterol, LDL‐C, and lipoprotein levels in hypercholesterolemic participants (Zambón et al. 2000). Adding *J. regia* kernel to the diet of outpatients with borderline high total cholesterol had advantageous effects on blood cardiovascular risk factors by increasing HDL‐C, tPA, and lessening triacyglycerols, total cholesterol, LDL‐C, and PAI‐1 (Morgan et al. 2002). Administering *J. regia* oil as a supplement to samples with hyperlipidemia led to a notable decrease in blood triglyceride levels (Zibaeenezhad et al. 2003). Likewise, consuming *J. regia* kernel decreased plasma triglyceride levels and enhanced HDL‐C amounts in hyperlipidemic individuals (Zibaeenezhad, Shamsnia, and Khorasani 2005). The administration of *J. regia* kernel to healthy subjects, enhanced HDL, lowered total cholesterol and LDL (Fitschen et al. 2011). The findings of another clinical trial disclosed that receiving *J. regia* leaf aqueous ethanolic extract in type II diabetic patients, decreased levels of total cholesterol and triglycerides (Hosseini et al. 2014). Moreover, taking *J. regia* kernel increased HDL‐C in hyperlipidemic patients (Tufail et al. 2015). It has been claimed that oil might modulate coronary artery disease risk factor hyperlipidemic type 2 diabetic patients by augmenting HDL amounts and lowering the levels of total cholesterol, triglyceride, LDL, and total cholesterol/HDL ratio (Zibaeenezhad et al. 2017). In another investigation, consumption of *J. regia* kernel by people with dyslipidemia resulted in the augmented amounts of HDL‐C, as well as declined levels of total cholesterol, triglyceride, LDL‐C, and very low‐density lipoprotein cholesterol (VLDL‐C) (Bashan and Bakman 2018). Consumption of *J. regia* kernel in postmenopausal women with hypercholesterolemia reduced VLDL and LDL levels (Borkowski et al. 2019). The administration of *J. regia* kernel to people with hyperlipidemia significantly increased HDL‐C and lessened total cholesterol, triglyceride, and LDL‐C (Ashraf et al. 2020; Table 2).

**TABLE 2:** The therapeutic effects of *J. regia* against hyperlipidemia.

Compound	Study design	Doses/Duration	Results	References
In vitro				
*J. regia* oil	HepG2 cells		↑Cholesterol efflux, ABCG1 gene expression ↓Cholesterol synthesis, CYP51, HMGCR, SREBP‐2 gene expression, TC, triacylglycerol	Gao et al. (2022b)
In vitro plus in vivo				
*J. regia* dried kernel pellicles polyphenol‐rich extract (50% ethanol)	HepG2 cells	*J. regia* polyphenol‐rich extract, 100 μg/mL, 24 h Tellimagrandin: 1–100 μg/mL	↑mRNA expressions of PPARα, ACOX1, CPT1A ↑ACOX1 expression	Shimoda et al. (2009)
	HFD‐fed male ddY mice	50, 100, 200 mg/kg, 13 days, p.o.	↑mRNA expressions of PPARα, ACOX1 ↓ TG	
In vivo				
*J. regia* leaf alcoholic extract	Wistar rats with type 1 diabetes	200, 400 mg/kg, i.g., 28 days	↑HDL‐C ↓LDL, TC, TG	Mohammadi et al. (2011)
Whole *J. regia*	Male Wistar diabetic rats	4%, 4 weeks, p.o.	↑HDL‐C, PPARα ↓Atherosclerosis risk factors, cholesterol, LDL‐C, VLDL‐C, TG, SREBP‐1c protein level	Ebrahim Abbasi, Arash Noori, and Ali (2012)
*J. regia* fruit internal septum ethanolic extract	Male Albino Wistar diabetic rats	0–400 mg/kg, 28 days, p.o.	↓ TG, TC, LDL	Ghiravani et al. (2016)
*J. regia* powder	HFD‐fed male Sprague Dawley rats	10%–40%	↑HDL‐C, blood anti‐oxidant capacity ↓LDL‐C	Abrar et al. 2019)
*J. regia* nut oil‐derived polyunsaturated fatty acid	Wistar diabetic pregnant rats	225, 450, 900 mg/kg, 17 days, gavage	↑HDL‐C ↓TC, TG, LDL‐C, SREBP‐1, SCD‐1, FAS, ACC	Sun et al. (2020)
*J. regia* leaves ethanolic extract	Male Sprague Dawley rats with HC	200 mg/kg, 4 weeks, gavage	↑HDL‐C ↓TG, LDL, atherogenic index	Azhar et al. (2021)
*J. regia* kernel meal peptides	HFD‐fed male Sprague Dawley rats	200, 400, 800 mg/kg, 4 weeks, p.o.	↑HDL‐C, LCAT, CYP7A1 ↓Atherosclerosis index, TC, TG, LDL‐C, Apo‐B, Apo‐A1, FAS, HMGR	Yang et al. (2021)
*J. regia* husk methanolic extract	Male Albino Wistar rats with HC	200, 400, 800 mg/kg, 84 days, p.o.	↑HDL ↓Cholesterol, LDL, TG	Rozha et al. (2021)
*J. regia* (whole fruit without shells)	Female Wistar Albino rats with HL	153 mg, 15 days, p.o.	↓ VLDL	Sangi, Shamim, and Khan (2022)
Defatted *J. regia* kernel powder extract	Male Sprague Dawley rats with hyperlipidemic acute pancreatitis	0.18, 0.36, 0.72 g/kg, 10 weeks, gavage	↓TC, TG, LDL‐C	Xu et al. (2022)
*J. regia* fruit diaphragm ethanolic extract	Male Wistar rats with HL	150, 300 mg	↑HDL‐C, CAT, SOD ↓TC, TG, MDA	Palabiyik et al. (2023)
Clinical trial				
*J. regia* (raw, shelled)	49 individuals with HC	41–56 g, 6 weeks, p.o.	↓TC, LDL‐C, lipoprotein	Zambón et al. (2000)
*J. regia* kernel	67 borderline high cholesterol outpatients	64 g, 6 weeks, p.o.	↑HDL‐C, tPA ↓ Triacyglycerols, TC, LDL‐C, PAI‐1	Morgan et al. (2002)
*J. regia* oil	60 subjects with HL	3 g, 45 days, p.o.	↓ TG	Zibaeenezhad et al. (2003)
*J. regia* kernel	52 individuals with HL	20 g, 8 weeks, p.o.	↑HDL‐C ↓ TG	Zibaeenezhad, Shamsnia, and Khorasani (2005)
*J. regia* kernel	36 participants	30 g, 30 days, p.o.	↑HDL ↓TC, LDL	Fitschen et al. (2011)
*J. regia* leaf aqueous ethanolic extract	61 diabetic patients	200 mg/day, 3 months, p.o.	↓TC, TG	Hosseini et al. (2014)
*J. regia* kernel	20 participants with HL	30 g, 8 weeks, p.o.	↑HDL‐C	Tufail et al. (2015)
*J. regia* oil	100 type 2 diabetes patients with HL	15 mL, 90 days, p.o.	↑HDL ↓TC, TG, LDL, total cholesterol/HDL ratio	Zibaeenezhad et al. (2017)
*J. regia* kernel	73 people with dyslipidemia	3 months, 40–50 g, p.o.	↑HDL‐C ↓ TC, TG, LDL‐C, VLDL‐C	Bashan and Bakman (2018)
*J. regia* kernel	15 postmenopausal women with HC	40 g, 4 weeks, p.o.	↓ VLDL, LDL	Borkowski et al. (2019)
*J. regia* kernel	90 subjects with HL	25, 50 g, 56 days, p.o.	↑HDL‐C ↓ TC, TG, LDL‐C	Ashraf et al. (2020)

Abbreviations: ABCG1, ATP‐binding cassette subfamily G member 1; ACC, Acetyl‐CoA carboxylase; ACOX1, Acyl‐CoA oxidase 1; Apo, Apolipoprotein; CAT, Catalase; CPT1A, Carnitine palmitoyltransferase 1A; CYP51, Cytochrome P450 51; CYP7A1, Cytochrome P450 7A1; FAS, Fatty acid synthase; HC, hypercholesterolemia, HDL‐C, High‐density lipoprotein cholesterol; HFD, high‐fat diet; HL, hyperlipidemia, HMGCR, 3‐hydroxy‐3‐methylglutaryl‐coenzyme A reductase; HMGR, Hydroxymethylglutaryl‐coenzyme A reductase; LCAT, Lecithin‐cholesterol acyltransferase; LDL‐C, Low‐density lipoprotein cholesterol; MDA, Malondialdehyde; mRNA, Messenger RNA; PAI‐1, Plasminogen activator inhibitor‐1; PPARα, Peroxisome proliferator‐activated receptor alpha; SCD‐1, Stearoyl‐CoA desaturase‐1; SOD, Superoxide dismutase; SREBP, Sterol regulatory element‐binding protein; TC, total cholesterol; TG, triglycerides; tPA, Tissue plasminogen activator; VLDL‐C, Very low‐density lipoprotein cholesterol.

**FIGURE 2:**
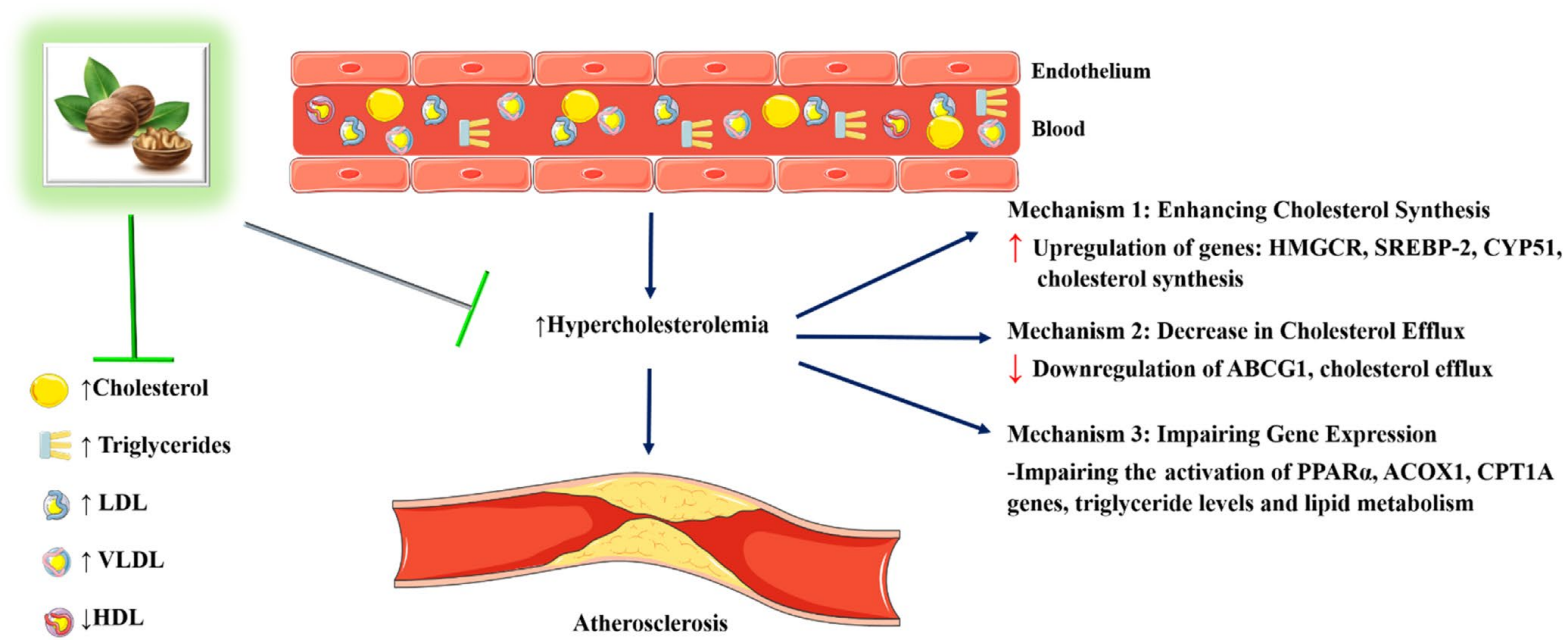
The ameliorative effect of *J. regia* on hyperlipidemia and atherosclerosis.

In summary, numerous studies have consistently demonstrated the potential benefits of *J. regia* in ameliorating hyperlipidemia and reducing the risk of cardiovascular disease (Figure 2). The underlying mechanisms behind these effects involve the modulation of lipid profiles and metabolism. The consumption of *J. regia* has been shown to lower cholesterol levels, including total cholesterol and LDL cholesterol, while increasing the levels of beneficial HDL cholesterol. *J. regia* has been found to affect various genes and proteins involved in lipid metabolism, such as downregulating the expression of genes like HMGCR, SREBP‐2, and CYP51, which are involved in cholesterol production. It also promotes cholesterol efflux by upregulating the expression of ABCG1, leading to decreased total cholesterol and triglyceride levels. Additionally, it upregulates the expression of genes related to fatty acid oxidation, such as PPARα, ACOX1, and CPT1A, resulting in improved lipid profiles.

Clinical trials have further supported the lipid‐lowering effects of *J. regia* in humans. Participants who incorporated *J. regia* into their diets experienced reductions in total cholesterol, LDL cholesterol, and lipoprotein levels, while HDL cholesterol levels increased. These trials have also shown beneficial effects on cardiovascular risk factors, such as decreased triacylglycerols, PAI‐1, and improved tPA levels. Overall, the studies highlight the potential of *J. regia* in improving lipid profiles and reducing the risk of cardiovascular disease. The underlying mechanisms involve the modulation of genes and proteins related to cholesterol synthesis, cholesterol efflux, and lipid metabolism. However, it is important to conduct further research, including long‐term clinical trials, to establish optimal dosage, duration, and potential interactions with other dietary factors.

### 3.2 Hypertension

Hypertension, also known as high blood pressure, is a chronic medical condition characterized by elevated blood pressure levels persistently exceeding the normal range (Jalali and Ghasemzadeh 2022). It is a prevalent cardiovascular risk factor affecting a significant portion of the global population. Hypertension is a major factor in the development and progression of CVDs such as coronary artery disease, stroke, and heart failure (Yahyazadeh et al. 2021; Naraki et al. 2023). Several factors contribute to the pathogenesis of hypertension and its association with CVDs.

Oxidative stress and inflammation are key processes involved in the pathogenesis of hypertension and its association with CVDs (Guzik and Touyz 2017). Oxidative stress leads to endothelial dysfunction, vascular inflammation, and increased blood pressure (Dinh et al. 2014). Glutathione peroxidase (GPx) and glutathione (GSH), components of the antioxidant defense system, help mitigate oxidative damage and maintain vascular health (Leopold and Loscalzo 2005). Inflammatory mediators, such as tumor necrosis factor‐alpha (TNF‐α), contribute to endothelial dysfunction, vascular inflammation, and elevated blood pressure (Theofilis et al. 2021). They promote the expression of adhesion molecules like vascular cell adhesion molecule‐1 (VCAM‐1), which facilitate immune cell adhesion and infiltration into the vessel walls (Troncoso et al. 2021; Singh et al. 2023).

Besides, nitric oxide (NO) is a crucial vasodilator that regulates blood pressure by promoting arterial relaxation (Kumar, Dey, and Kundu 2020). Reduced NO bioavailability, often associated with oxidative stress and inflammation, leads to increased vascular tone and elevated blood pressure (Crosswhite and Sun 2010; Griendling et al. 2021). Furthermore, within the renin–angiotensin–aldosterone system, angiotensin II, generated by ACE from angiotensin I, acts as a potent vasoconstrictor, promoting hypertension (Ahmad et al. 2023). Angiotensin II also stimulates the release of aldosterone, which leads to sodium and water retention, further contributing to increased blood pressure (Gasparini et al. 2019). Additionally, endothelin‐1, a potent vasoconstrictor, plays a role in hypertension by promoting increased vascular resistance and elevated blood pressure (Kostov 2021).

The following section will delve into the research conducted on the effect of *J. regia* on hypertension.

#### 3.2.1 In Vivo

An in vivo study aimed to investigate the properties and health benefits of *J. regia* protein and its enzymatic hydrolysates. The *J. regia* protein was hydrolyzed using alcalase and trypsin, resulting in alcalase‐generated *J. regia* protein hydrolysate and trypsin‐generated *J. regia* protein hydrolysate. The hydrolysates were compared in terms of physico‐chemical properties, antioxidant activity, ACE inhibitory activity, and stability. The *J. regia* protein hydrolysate exhibited superior physico‐chemical properties, antioxidant activity, ACE inhibitory activity, and stability against thermal treatment and gastrointestinal digestion compared to the *J. regia* protein. Additionally, the antihypertensive effects of the hydrolysates were evaluated in spontaneously hypertensive rats. Both alcalase‐generated *J. regia* protein hydrolysate and trypsin‐generated *J. regia* protein hydrolysate showed significant reductions in systolic blood pressure, with the most potent effects observed at 4 and 6 h after administration, respectively. The study concluded that *J. regia* protein hydrolysate demonstrated excellent properties, potent inhibitory activities, and high stability, with trypsin‐generated *J. regia* protein hydrolysate showing greater effectiveness than alcalase‐generated *J. regia* protein hydrolysate (Wang et al. 2016). It has been shown that the administration of a methanolic extract of *J. regia* kernel to hypertensive rats prohibited diastolic hypertension by increasing GPx levels and serum NO levels. It also reduced the MDA/GPx ratio (Joukar et al. 2017). Receiving *J. regia* kernel in mice with metabolic syndrome could pointedly decrease mean arterial pressure (MAP) (Scott et al. 2017). The supplementation of *J. regia* seeds oil to hypertensive rats resulted in reduced blood pressure through increasing the amounts of NO, GSH, and attenuating ACE, endothelin‐1, and MDA (Mohammed and Hussein 2020). Another study investigated the role of *J. regia* protein enzymatic hydrolysis in exerting ACE inhibitory activity. In vitro simulated digestion showed that *J. regia* protein could be effectively digested by pepsin and pancreatin, resulting in ACE inhibitory activity comparable to that of *J. regia* protein hydrolysate. In vivo experiments demonstrated that both *J. regia* protein and *J. regia* protein hydrolysate significantly reduced systolic blood pressure in acute and long‐term oral administration. Furthermore, *J. regia* protein inhibited ACE activity in the aorta, kidney, and lung tissues of spontaneously hypertensive rats, along with a decrease in levels of endothelin‐1 and TNF‐α, and an increase in levels of bradykinin and NO in the serum. Similarly, *J. regia* protein hydrolysate reduced blood pressure by inhibiting ACE activity in spontaneous hypertension rats' aorta and lung tissues, lowering TNF‐α levels, and increasing bradykinin and NO levels in the serum. In summary, *J. regia* protein exhibited a more stable and longer‐lasting antihypertensive effect compared to *J. regia* protein hydrolysate (Liu, Guo, et al. 2021). The administration of two peptides purified and isolated from defatted *J. regia* meal hydrolysates (VERGRRITSV and FVIEPNITPA) to hypertensive rats attenuated their systolic and diastolic pressures by reducing angiotensinogen, ACE, angiotensin II, aldosterone, endothelin‐1, and enhancing the amounts of serum NO (Wang et al. 2021).

#### 3.2.2 Clinical Trial

*Juglans regia* kernel consumption remarkably boosted endothelium‐dependent vasodilation and declined VCAM‐1 levels in hypercholesterolemic individuals (Ros et al. 2004). It has been disclosed that adding alpha‐linolenic acid and linoleic acid to the foods of hypercholesterolemic subjects consuming the average American diet lessened total peripheral resistance and diastolic blood pressure that were evident during stress and at rest. Flow‐mediated dilation is amplified by receiving additional alpha‐linolenic acid. Moreover, arginine‐vasopressin increased, but endothelin‐1 was unchanged. The authors stated that the slight rise in arginine‐vasopressin could be a reaction to lowered blood pressure and could also explain the notable increase in stroke volume observed during the intervention diets (West et al. 2010). A hydroalcoholic extract of *J. regia* leaves attenuated systolic blood pressure in diabetic patients (Rabiei et al. 2018). Consuming *J. regia* kernel could reduce systolic blood pressure in people with mild hypertension (Domènech et al. 2019). Adding *J. regia* kernel to the diet of elderly participants could decrease their systolic and diastolic pressure, although it was not significant (Al Abdrabalnabi et al. 2020; Table 3).

**TABLE 3:** The therapeutic effects of *J. regia* against hypertension.

Compound	Study design	Doses/Duration	Results	References
In vivo				
*J. regia* kernel methanolic extract	Male Wistar rats with HT	100, 200 mg/kg, 15 days, gavage	Prohibited diastolic hypertension ↑GPx level, serum NO level ↓ MDA/GPx ratio	Joukar et al. (2017)
*J. regia* kernel	Mice with metabolic syndrome	0.0125 g/mouse/day, 21 weeks, p.o.	↓ MAP	Scott et al. (2017)
*J. regia* seeds oil	Male Albino rats with HT	3 mL/kg, 3 weeks, gavage	↑NO, GSH ↓ BP, ACE, endothelin‐1, MDA	Mohammed and Hussein (2020)
*J. regia* protein, *J. regia* protein hydrolysate	Rats with HT	*J. regia* protein: 400, 800 mg/kg, 4 weeks, i.g. *J. regia* protein hydrolysate: 800 mg/kg, 4 weeks, i.g.	Inhibited ACE activity in aorta, kidney, lung tissues ↑Bradykinin, NO ↓ BP, endothelin‐1, TNF‐α	Liu, Guo, et al. (2021)
ERGRRITSV and FVIEPNITPA peptides isolated from *J. regia* meal hydrolysates	Male spontaneously hypertensive rats	5, 15 mg/kg, 4 weeks, p.o.	↑Serum NO ↓ SBP, DBP, angiotensinogen, ACE, Angiotensin II, aldosterone, endothelin‐1	Wang et al. (2021)
Clinical trial				
*J. regia* kernel	21 hypercholesterolemic individuals	40–65 g/day, 4 weeks, p.o.	↑Endothelium‐dependent vasodilation ↓ VCAM‐1	Ros et al. (2004)
Alpha‐linolenic acid, linoleic acid	20 hypercholesterolemic individuals	37 g *J. regia*, 15 g *J. regia* oil, 18 weeks, p.o.	↑Flow mediated dilation, arginine‐vasopressin ↓ DBP, total peripheral resistance	West et al. (2010)
*J. regia* leaves hydroalcoholic extract	40 diabetic patients	100 mg, twice a day, 8 weeks, p.o.	↓ SBP	Rabiei et al. (2018)
*J. regia* kernel	236 individuals with mild hypertension	15% energy, 2 year, p.o.	↓ SBP	Domènech et al. (2019)
*J. regia* kernel	625 elderly participants	1, 1.5, 2 oz., 2 years, p.o.	↓ SBP, DBP	Al Abdrabalnabi et al. (2020)

Abbreviations: ACE, Angiotensin‐converting enzyme; BP, blood pressure; DBP, diastolic blood pressure; GPx, glutathione peroxidase; GSH, glutathione; HT, Hypertension; MAP, mean arterial pressure; MDA, malondialdehyde; NO, nitric oxide; SBP, systolic blood pressure; TNF‐α, tumor necrosis factor‐alpha; VCAM‐1, vascular cell adhesion molecule‐1.

**FIGURE 3:**
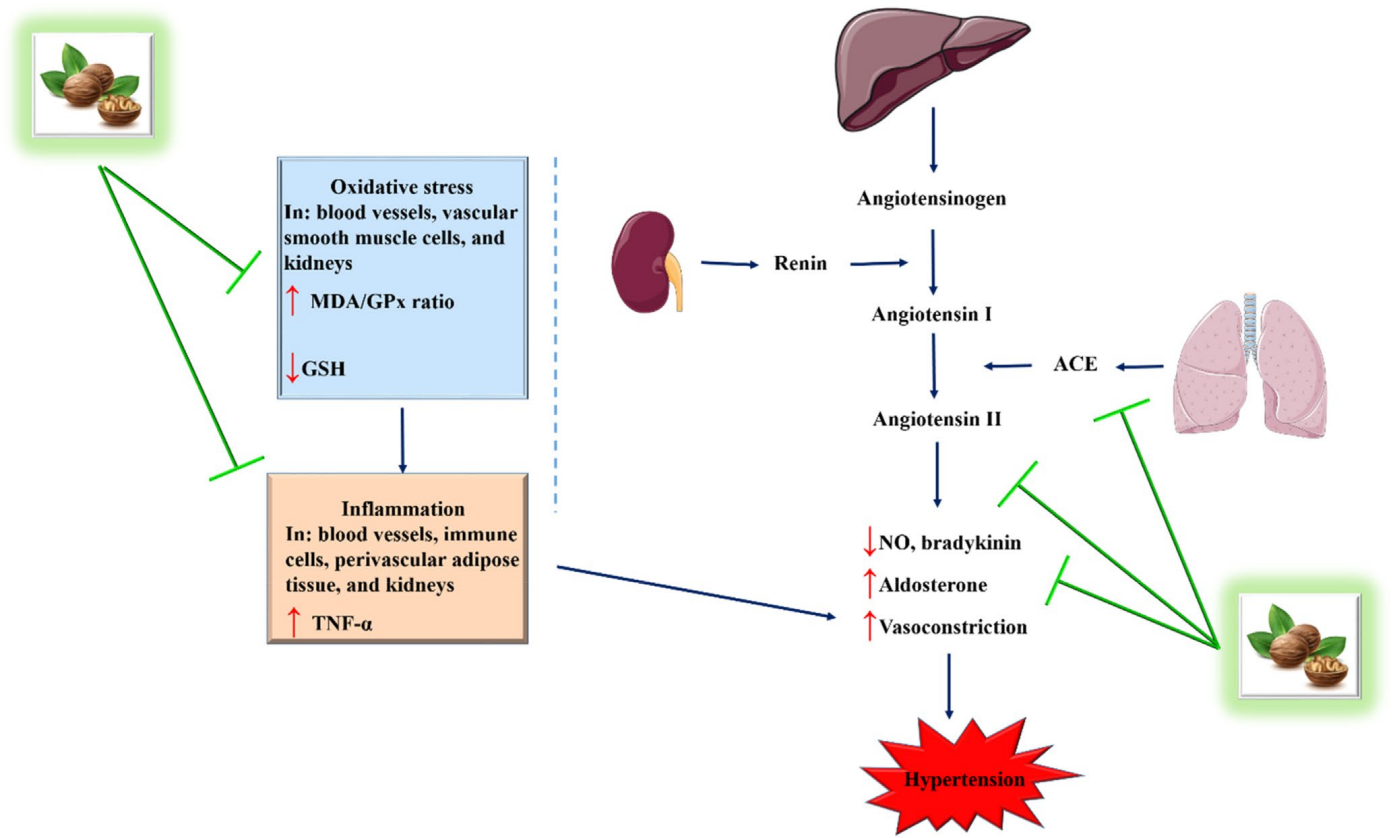
The ameliorative effect of *J. regia* on hypertension.

To sum up, the in vivo study and clinical trials on *J. regia* protein and its hydrolysates have demonstrated their potential in regulating blood pressure and cardiovascular health (Figure 3).

The contradiction observed in the results of Wang et al. (2016) and Liu, Guo, et al. (2021) can be explained as follows: Wang et al. (2016) study highlights that walnut protein hydrolysates exhibited superior physico‐chemical properties, antioxidant activity, and ACE inhibitory effects compared to walnut protein. The significant reduction in systolic blood pressure observed after administering walnut protein hydrolysates to spontaneously hypertensive rats suggests its potent antihypertensive effects, particularly at 4 h (−26 mmHg) and 6 h (−30 mmHg) post‐administration. This study emphasizes the potential benefits of walnut protein hydrolysates in reducing blood pressure and underlines its high stability against various conditions. On the other hand, Liu, Guo, et al. (2021) focuses on the role of enzymatic hydrolysis on ACE inhibitory activity. The study demonstrates that both walnut protein and walnut protein hydrolysates effectively reduce systolic blood pressure, with walnut protein showing a more stable and longer‐lasting antihypertensive effect compared to walnut protein hydrolysates. The in vivo results indicate that walnut protein and walnut protein hydrolysates significantly reduced blood pressure, with walnut protein exhibiting a slightly greater reduction in systolic blood pressure in long‐term intragastric administration. The contradiction between the two references could stem from various factors such as the specific enzymatic hydrolysis methods used, differences in peptide profiles between walnut protein and walnut protein hydrolysates, variations in experimental protocols, or the presence of bioactive components that may influence blood pressure regulation differently in each study. These contrasting findings underscore the complexity of bioactive interactions and the need for further research to elucidate the mechanisms behind the observed effects of walnut protein and its hydrolysates on blood pressure regulation.

In general, the antihypertensive effects were attributed to the inhibition of ACE activity, which led to increased levels of bradykinin and NO, and decreased levels of endothelin‐1 and pro‐inflammatory markers. Additionally, *J. regia* consumption showed improvements in endothelium‐dependent vasodilation, reduced VCAM‐1 levels, and decreased peripheral resistance. These findings suggest that *J. regia* and its components hold promise as natural resources for developing functional foods or nutraceuticals targeting hypertension and cardiovascular health, with underlying mechanisms involving ACE inhibition, modulation of vasoactive substances, and antioxidant effects. Further research is needed to fully explore the potential of *J. regia* in promoting human health.

### 3.3 Heart Failure and Myocardial Infarction

Heart failure and myocardial infarction are significant components of CVDs (Fathima 2021). Heart failure occurs when the heart's ability to pump blood efficiently is compromised, leading to inadequate oxygen and nutrient supply to the body's organs and tissues. It can develop due to various underlying conditions, including hypertension, coronary artery disease, or previous myocardial infarction (Schwinger 2021).

A myocardial infarction, commonly known as a heart attack, is characterized by a sudden interruption of blood flow to a part of the heart muscle. This interruption is typically caused by a blockage in one of the coronary arteries (Saleh and Ambrose 2018). The blockage restricts oxygen supply to the affected area, resulting in irreversible damage to the heart tissue (Basalay, Yellon, and Davidson 2020).

Several factors play crucial roles in the development and progression of heart failure and myocardial infarction. Oxidative damage is associated with both conditions through cellular dysfunction, inflammation, and damage to the heart tissue (van der Pol et al. 2019). Dyslipidemia is another contributing factor, and high levels of LDL‐C and low levels of HDL‐C are particularly associated with an increased risk of developing atherosclerosis and potentially leading to myocardial infarction (Sun et al. 2022; Zhong et al. 2019). Additionally, the expression of NADPH oxidase (NOX)4, a key enzyme involved in the production of ROS, has been implicated in the pathogenesis of heart failure and myocardial infarction (Stevenson et al. 2019). Increased NOX4 expression contributes to oxidative stress and inflammation in the cardiovascular system, further exacerbating the damage to the heart tissue (Vendrov et al. 2023). In the upcoming section, we will report the studies carried out to investigate the impact of *J. regia* on heart failure and/or myocardial infarction.

#### 3.3.1 In Vivo

It has been disclosed that *J. regia* kernel extracts prohibited myocardial infarction in rats exposed to isoproterenol by reducing dyslipidemia and oxidative damage (Sun et al. 2019). Another study aimed to investigate the effects of *J. regia* supplementation on the silent information regulator 1 (SIRT1)/forkhead box O3a (FoxO3a)/manganese superoxide dismutase (MnSOD)/catalase axis, and other molecular markers in the heart in the context of a fructose‐rich diet. The findings of the study revealed that *J. regia* supplementation reversed the unfavorable changes induced by the fructose‐rich diet in the SIRT1/FoxO3a/MnSOD/catalase axis in the heart. Interestingly, both the fructose‐rich diet and *J. regia* supplementation led to an increase in NOX4 expression. The fructose‐rich diet resulted in a shift in the carbohydrate response element‐binding protein (ChREBP) distribution, increasing its cytosolic fraction and decreasing its nuclear fraction in the heart. In control rats supplemented with *J. regia*, the nuclear fraction of ChREBP decreased. Moreover, *J. regia* kernel consumption was associated with a reduction in systolic blood pressure in fructose‐rich diet‐fed rats and a decrease in fatty acid arachidonic acid/eicosapentaenoic acid and arachidonic acid/docosahexaenoic acid ratios in plasma (Bošković et al. 2021). A study aimed to investigate the cardioprotective effects of juglone, a major component of the walnut tree, using a rat model of isoproterenol‐induced myocardial infarction. The results of the study demonstrated that pretreatment with juglone showed cardioprotective effects by reducing myocardial damage induced by isoproterenol. This was evidenced by improvements in electrocardiogram (ECG) parameters such as reduced T‐waves and deep Q‐waves. Additionally, juglone administration resulted in a reduction in heart‐to‐body weight ratio, infarction size, and normalization of cardiac marker enzymes, including cardiac troponin I (cTnI), creatine phosphokinase (CPK), creatine kinase‐MB (CK‐MB), lactate dehydrogenase (LDH), alanine aminotransferase (ALT), and aspartate aminotransferase (AST). Histopathological examination revealed a decrease in inflammation, edema, and necrosis in the myocardial tissue (Ahmad et al. 2022). The results of another study revealed that the administration of *J. regia* oil to rats showed a negative troponin, a non‐significant decrease in mean serum CK‐MB levels, and a non‐significant increase in mean serum vitamin D levels in the treated group compared to the control group (Rajab et al. 2022).

#### 3.3.2 Clinical Trial

These findings from a clinical trial indicated that a higher consumption of alpha‐linolenic acid in the diet could potentially lower the chances of experiencing sudden cardiac death among women. However, there is no evidence to suggest a similar effect on other types of fatal coronary heart disease or nonfatal myocardial infarction in women (Albert et al. 2005; Table 4).

**TABLE 4:** The therapeutic effects of *J. regia* against heart failure and myocardial infarction.

Compound	Study design	Doses/Duration	Results	References
In vivo				
*J. regia* kernel extracts	Male Sprague–Dawley rats with MI	300 mg/kg, 35 days, p.o.	Prohibited myocardial injury ↓ Oxidative damage and dyslipidemia	Sun et al. (2019)
*J. regia* kernel	FRD‐fed male Wistar rats	2.4 g, 6 weeks, p.o.	↑NOX4 expression ↓ Nuclear fraction of ChREBP, SBP, fatty acid arachidonic acid/eicosapentaenoic acid, arachidonic acid/docosahexaenoic acid ratios in plasma	Bošković et al. (2021)
Juglone	Sprague–Dawley Rats with MI	1, 3 mg/kg, 5 days, i.p.	Fortified the myocardial cell membrane Prevented infarction, elevated T‐waves, deep Q‐waves in the ECG Normalized cTnI, CPK, CK‐MB, LDH, ALT, AST ↓ Extent of myocardial damage, histopathological changes	Ahmad et al. (2022)
*J. regia* oil	Healthy male Albino rats		↑Mean serum vitamin D levels ↓ Serum CK‐MB levels	Rajab et al. (2022)
Clinical trial				
Alpha‐Linolenic Acid	76,763 women participating in the Nurses' Health Study		↓ Risk of sudden cardiac death	Albert et al. (2005)

Abbreviations: ALT, alanine aminotransferase; AST, aspartate aminotransferase; ChREBP, carbohydrate response element‐binding protein; CK‐MB, creatine kinase‐MB; CPK, creatine phosphokinase; cTnI, cardiac troponin I; ECG, electrocardiogram; FRD, fructose‐rich diet; LDH, lactate dehydrogenase; MI, myocardial infarction; SBP, systolic blood pressure.

Briefly, research on *J. regia* and its constituent parts has demonstrated encouraging cardioprotective properties against myocardial infarction and heart failure. They have revealed mechanisms such as reducing dyslipidemia, oxidative damage, and myocardial damage. *J. regia* supplementation has been associated with positive changes in cardiac parameters, including troponin levels and serum CK‐MB. Additionally, a higher consumption of alpha‐linolenic acid from *J. regia* may lower the risk of sudden cardiac death in women. However, further study is required to completely understand and confirm these findings as well as to investigate the potential therapeutic uses of *J. regia* for cardiovascular health.

### 3.4 Platelet Aggregation and Atherosclerosis

Atherosclerosis and platelet aggregation are important components of CVDs (Khodadi 2020). Platelet clumping and blood clot formation are referred to as platelet aggregation (Ye et al. 2020), whereas the accumulation of fatty plaques in arteries is known as atherosclerosis (Babaniamansour et al. 2020). The production of clots is facilitated by platelet aggregation and can obstruct blood flow to vital organs (Sang et al. 2021). Atherosclerosis can lead to plaque rupture that results in clot formation, narrowing arteries, and restricting blood flow. These processes contribute to conditions like heart attacks, strokes, and peripheral arterial disease, highlighting their significance in CVD development (Bentzon et al. 2014).

Several variables contribute to platelet aggregation. ROS promote platelet activation and aggregation, resulting in an ideal condition for clot formation. It can also enhance platelet adhesion to the vascular wall and promote platelet hyperreactivity (Masselli et al. 2020). NADPH Oxidase 2 (NOX2), an enzyme responsible for ROS production, is involved in platelet activation and vascular dysfunction, further exacerbating platelet aggregation (Sonkar et al. 2019). Endothelial nitric oxide synthase (eNOS) and its product, NO, play a counteractive role against platelet aggregation (Emami et al. 2019). NO prevents excessive clot formation by inhibiting platelet adhesion and aggregation. Furthermore, NO stimulates vasodilation and preserves endothelial function, assisting to reduce platelet aggregation and atherosclerosis (Gori 2020). Platelet aggregation and atherosclerosis are also influenced by inflammation (Marchio et al. 2019). Interleukin‐1β (IL‐1β) is a pro‐inflammatory cytokine that can activate and aggregate platelets, increasing the risk of clot formation. IL‐1β is also linked to immune cell recruitment and the inflammation found in atherosclerotic plaques (Beaulieu et al. 2014). Monocyte chemoattractant protein‐1 (MCP‐1) plays a role in this process by attracting monocytes to sites of vascular inflammation, impairing plaque instability, and promoting platelet activation (Rayes et al. 2020; Mebrat 2023).

Furthermore, the transcription factor Krüppel‐like Factor 2 (KLF2) protects against platelet aggregation and atherosclerosis (Dabravolski et al. 2022). KLF2 promotes endothelial integrity and serves by increasing the expression of genes involved in vascular homeostasis and decreasing platelet activation (Xu et al. 2021). Protein kinase B (Akt), a signaling molecule, also contributes to platelet aggregation and atherosclerosis (Song et al. 2019). Endothelial function is regulated by Akt activation pathways, which enhance cell survival and inhibit apoptosis (Jing et al. 2019). Intracellular calcium ions (Ca^2+^) are essential for platelet aggregation. Elevated intracellular Ca^2+^ levels activate platelets and increase the release of clot‐forming components (Back et al. 2022). Ca^2+^ signaling is also involved in endothelial cell function, vascular smooth muscle contraction, and cardiac muscle contraction, all of which affect platelet aggregation and atherosclerosis (Filippini, D'Amore, and D'Alessio 2019; Negri, Faris, and Moccia 2021).

In the following section, we will discuss the research conducted on the effects of *J. regia* on platelet aggregation and atherosclerosis.

#### 3.4.1 In Vitro

It has been reported that treating human blood platelets exposed to adenosine diphosphate with a polyphenolic extract of *J. regia* husks causes a reduction in platelet aggregation in whole blood, platelet reactivity index, and platelet hyper‐reactivity (Rywaniak et al. 2015). In an in vitro study, the researchers aimed to assess the content and biological effects of *J. regia* hull extract on platelet function. The experimental data revealed that the acetone extract of *J. regia* hulls contained a high amount of polyphenolic compounds and exhibited antioxidant properties. Furthermore, *J. regia* hull extract inhibited thrombin‐induced platelet aggregation and protein secretion without causing any cytotoxic effects on platelets. The extract also suppressed the generation of ROS and the activation of caspase in thrombin‐stimulated platelets (Meshkini and Tahmasbi 2017). Treating human aortic endothelial cells exposed to oscillatory shear stress with juglanin, a natural phenolic compound, resulted in reduced oxidative stress by decreasing the production of ROS. This effect was achieved through the downregulation of NOX2 and the restoration of oscillatory shear stress‐induced reduced expression of eNOS. Furthermore, juglanin exhibited anti‐inflammatory properties by suppressing the oscillatory shear stress‐induced expressions of IL‐1β, MCP‐1, and high mobility group box 1 (HMGB1). The study also revealed that juglanin inhibited the attachment of monocytes to endothelial cells by reducing the expression of VCAM‐1 and E‐selectin. Besides, juglanin rescued the oscillatory shear stress‐induced reduction in the expression of KLF2 (Zhao et al. 2020). Exposing platelets to juglone resulted in the inhibition of platelet aggregation and the activation of glycoprotein IIb/IIIa induced by various agonists. Furthermore, in a whole blood flow chamber system, juglone demonstrated the ability to reduce thrombus formation on collagen‐coated surfaces, particularly under arterial shear rates. The researchers also found that juglone effectively abolished the elevation of intracellular Ca^2+^ and the activation of protein kinase C induced by collagen. However, it did not significantly impact the Ca^2+^ elevation and protein kinase C activation caused by agonists that act through G protein‐coupled receptors. In contrast, juglone inhibited the activation of Akt induced by various agonists in platelets (Kao et al. 2021).

#### 3.4.2 Studies Including Both In Vitro and In Vivo Sections

The findings of another study revealed that the crude aqueous extract of *J. regia* root bark exhibited potent antiaggregant effects. In vitro experiments showed that the extract strongly inhibited platelet aggregation induced by adenosine diphosphate. Furthermore, the extract demonstrated anticoagulant effects by significantly prolonging the thrombin time and reducing fibrinogen levels. Notably, these effects did not interfere with the activated partial thromboplastin time or prothrombin time. Additionally, the crude aqueous extract of *J. regia* root bark was found to increase bleeding time in mice and rats (Amirou et al. 2018).

#### 3.4.3 In Vivo

In a study comparing the effects of feeding mice with *J. regia* and *J. regia* oil, it was found that feeding mice with *J. regia* significantly reduced atherosclerotic plaque development in the aortic arch compared to the control diet. This reduction was associated with decreased staining of plaques for CD36, a receptor expressed by macrophages. Additionally, mice fed with *J. regia* showed lower levels of prothrombin, cholesterol, and triglycerides. Furthermore, the accumulation of lipids in the liver decreased, and plasma antioxidant capacity increased. On the other hand, feeding mice with *J. regia* oil did not lead to significant changes in these parameters. Moreover, platelet activation and thrombus formation under flow remained unchanged with either the *J. regia* or *J. regia* oil diet (Nergiz‐Ünal et al. 2013). Supplementing mice on a high‐fat diet with *J. regia* seeds reduced platelet shape change and increased platelet count. There was also a trend towards increased bleeding time (Prabhu et al. 2016). Flavonoids extracted from *J. regia* demonstrated significant antithrombotic activity. They protected against paralysis or death induced by collagen and epinephrine, inhibited platelet aggregation, and prolonged plasmatic coagulation times (Amirou et al. 2022; Table 5).

**TABLE 5:** The therapeutic effects of *J. regia* against platelet aggregation and atherosclerosis.

Compound	Study design	Doses/Duration	Results	References
In vitro				
*J. regia* husks polyphenolic extract	Human blood platelets	7.5, 15 mg/m, 15 min	↓ Platelet aggregation in whole blood, platelet reactivity index, platelet hyper‐reactivity	Rywaniak et al. (2015)
*J. regia* hull	Human blood platelets	25–200 μg/mL, 2 h	↓ Thrombin‐induced platelet aggregation and protein secretion, ROS production, caspase activity	Meshkini and Tahmasbi (2017)
Juglanin	Human aortic endothelial cells	2.5, 5 μM, 24 h	↓ Oxidative stress, ROS, NOX2, expression of HMGB1, MCP‐1, IL‐1β, VCAM‐1, E‐selectin	Zhao et al. (2020)
Juglone	Human blood platelets	1–5 μM, 3 min	Prevented platelet aggregation, activation of glycoprotein IIb/IIIa, Akt ↓ Thrombus formation, intracellular Ca^2+^, activation of protein kinase C	Kao et al. (2021)
In vitro plus in vivo				
*J. regia* root bark crude aqueous extract	Platelets	0.25, 0.5, 1 mg/mL, 5 min	↓ Platelet aggregation	Amirou et al. (2018)
	Healthy Wistar rats and albino mice	1, 1.5 g/kg, p.o., single dose	↑Bleeding time	
In vivo				
*J. regia*	HFD‐fed pathogen free Apoe^−/−^ male mice	1.2 g/5 g diet, 8 weeks, p.o.	No significant effect on platelet activation and thrombus formation ↑Plasma antioxidant capacity ↓ Atherosclerotic plaque development, cholesterol, TG, prothrombin	Nergiz‐Ünal et al. (2013)
*J. regia* seed	C57BL/6J male mice with normal and high fat simple carbohydrate diet	5 months	↑Platelet count, bleeding time	Prabhu et al. (2016)
Flavonoids isolated from *J. regia* root bark	Mice and Wistar rats with APT	250 mg/kg, 7 days, p.o.	Prevented platelet aggregation ↑Plasmatic coagulation times ↓ Paralysis or death	Amirou et al. (2022)

Abbreviations: APT, acute pulmonary thromboembolism; HFD, high‐fat diet; HMGB1, high mobility group box 1; IL‐1β, interleukin‐1β; MCP‐1, monocyte chemoattractant protein‐1; NOX2, NADPH oxidase 2; ROS, reactive oxygen species; VCAM‐1, vascular cell adhesion molecule 1; TG, triglycerides.

In brief, studies investigating the effects of *J. regia* extracts and its components on platelet function and atherosclerosis have provided promising findings. *J. regia* revealed its ameliorative properties through multiple mechanisms, such as inhibiting platelet aggregation, reducing ROS, and exhibiting antioxidant properties. The *J. regia* extracts also showed anti‐inflammatory effects and inhibited monocyte attachment to endothelial cells. Moreover, *J. regia* extracts exhibited antiplatelet and anticoagulant effects through prolonging thrombin time and increasing bleeding time, improving lipid profiles. Additionally, flavonoids extracted from *J. regia* demonstrated significant antithrombotic activity. Generally, these findings suggest that *J. regia* extracts and compounds may have therapeutic potential in preventing thrombotic events and improving cardiovascular health. However, further research is needed to explore the underlying mechanisms and evaluate their effectiveness and safety in clinical settings.

## 4 Future Developments

Several areas of research show promise for future expansion as the field of nutritional interventions for cardiovascular disorders. Addressing these difficulties would allow researchers to get a better knowledge of the role of ingesting *J. regia* in cardiovascular health, as well as its therapeutic potential.

One essential direction for future study is to understand the underlying processes by which *J. regia* exerts its cardioprotective properties. Despite the rising body of data supporting the cardiovascular advantages of *J. regia*, further research is needed to identify the particular cellular and molecular processes involved. Understanding these mechanisms will facilitate the optimization of therapeutic strategies and potentially lead to the development of targeted interventions.

Also, regarding the variety in study designs observed in the literature reviewed, including changes in walnut treatments, dosages, and durations, it is critical to consider the potential effects of these differences on the overall findings and their relevance. These variations not only highlight the importance of consistent techniques in future research but also call for a critical evaluation of the reported outcomes' comparability and generalizability. Addressing these discrepancies in study parameters is critical for improving the reliability and consistency of results drawn about the nutritional advantages of walnuts in cardiovascular health.

Furthermore, establishing accurate dose–response correlations between walnut intake and cardiovascular health is another critical subject for future research. Determining the proper dose of *J. regia* required for maximum benefits and investigating potential differences based on individual variables such as age, gender, and metabolic state can give useful information. This knowledge will be utilized to offer *J. regia* consumption recommendations and personalize dietary treatments to individual needs. Besides, investigating the synergistic effects of *J. regia* in combination with other dietary and lifestyle interventions is an area of great interest. Research should explore whether incorporating *J. regia* into a comprehensive cardiovascular disease prevention can enhance overall effects and provide additive or synergistic benefits beyond individual interventions. Recognizing the potential synergies will help refine dietary recommendations and optimize treatment strategies.

Although multiple studies have shown the short‐term effects of *J. regia* consumption on cardiovascular health, the long‐term impact has yet to be fully understood. Large‐scale, controlled clinical trials with long follow‐up periods are required to assess the long‐term benefits, safety, and potential risks of regular *J. regia* consumption. These experiments will provide useful information about the optimal duration and frequency of *J. regia* consumption.

Moreover, the emerging field of nutrigenomics offers exciting opportunities to explore the interactions between genes, diet, and cardiovascular health. Future research should investigate how individual genetic profiles influence the response to *J. regia* consumption and whether personalized nutrition approaches can optimize the cardiovascular advantages. Integrating genetic information with dietary recommendations may result in more targeted and effective therapies.

Additionally, it is essential to investigate the potential of *J. regia* and its main components as functional foods or nutraceuticals for cardiovascular disease prevention. Investigating the development of innovative food products or supplements supplemented with *J. regia* extracts or chemicals may improve bioavailability and efficacy, providing novel approaches for cardiovascular disease prevention and management. Considering the complex nature of dietary patterns, it is important to search potential interactions between *J. regia* and other dietary factors. Future research should investigate if combining *J. regia* with certain nutrients or dietary components might improve their cardiovascular benefits or have synergistic effects. Identifying these relationships can provide more comprehensive dietary recommendations.

In summary, investigations into the underlying mechanisms, the establishment of dose–response relationships, the examination of synergistic effects, the execution of long‐term clinical trials, the exploration of personalized nutrition approaches, the development of functional foods or nutraceuticals, and the comprehension of interactions with other dietary factors should be the main goals of future research endeavors. By advancing our understanding of *J. regia* intake in cardiovascular diseases, these efforts will open the door to better clinical results and dietary strategies that are optimized.

## 5 Conclusion

In conclusion, the comprehensive review of numerous studies supports the potential nutritional advantages of walnut (*Juglans regia* L.) for cardiovascular diseases. The consumption of *J. regia* has consistently shown positive effects on lipid profiles and metabolism, resulting in a reduction of total cholesterol and LDL‐C levels, an increased HDL‐C level, and improved gene regulation related to cholesterol synthesis and fatty acid oxidation. Furthermore, *J. regia* protein and its hydrolysates have demonstrated promising results in regulating blood pressure and cardiovascular health. The hydrolysates exhibit superior properties and ACE inhibitory activity, contributing to their antihypertensive effects. Besides, *J. regia* consumption improves endothelium‐dependent vasodilation, reduces VCAM‐1 levels, and decreases peripheral resistance, indicating its potential as a natural resource for functional foods or nutraceuticals targeting hypertension. Similarly, *J. regia* has shown cardioprotective properties against myocardial infarction and heart failure by reducing dyslipidemia, oxidative damage, and myocardial damage. Supplementation with *J. regia* has been associated with positive changes in cardiac parameters and a potential lower risk of sudden cardiac death. Additionally, studies focusing on platelet function and atherosclerosis have revealed the ameliorative properties of *J. regia* extracts and compounds. They inhibit platelet aggregation, reduce ROS, exhibit anti‐inflammatory effects, and demonstrate antiplatelet, anticoagulant, and antithrombotic activities. However, further research, including long‐term clinical trials, is necessary to establish optimal dosage, duration, and potential interactions with other dietary factors. Continued investigation will help fully explore the therapeutic uses of *J. regia* and its constituents in cardiovascular diseases.

## Author Contributions

**Mostafa Rashki:** writing – original draft (equal). **Mahboobeh Ghasemzadeh Rahbardar:** writing – original draft (equal), writing – review and editing (equal). **Mohammad Hossein Boskabady** conceptualization and supervision.

## Ethics Statement

The authors have nothing to report.

## Consent

The authors have nothing to report.

## Conflicts of Interest

The authors declare no conflicts of interest.

## Data Availability

No new data were created or analyzed during this study. Data sharing is not applicable to this article.
